# Histogram clustering for rapid time-domain fluorescence lifetime image analysis

**DOI:** 10.1364/BOE.427532

**Published:** 2021-06-21

**Authors:** Yahui Li, Natakorn Sapermsap, Jun Yu, Jinshou Tian, Yu Chen, David Day-Uei Li

**Affiliations:** 1 Key Laboratory of Ultra-fast Photoelectric Diagnostics Technology, Xi’an Institute of Optics and Precision Mechanics, Xi’an Shaanxi 710049, China; 2 Collaborative Innovation Center of Extreme Optics, Shanxi University, Taiyuan Shanxi 030006, China; 3 Department of Physics, Scottish Universities Physics Alliance, University of Strathclyde, Glasgow, G4 0NG, United Kingdom; 4 Strathclyde Institute of Pharmacy and Biomedical Sciences, University of Strathclyde, Glasgow G4 0RE, United Kingdom; 5 Department of Biomedical Engineering, University of Strathclyde, Glasgow G1 0NW, United Kingdom; 6 yahuili777@hotmail.com; 7 David.Li@strath.ac.uk

## Abstract

We propose a histogram clustering (HC) method to accelerate fluorescence lifetime imaging (FLIM) analysis in pixel-wise and global fitting modes. The proposed method’s principle was demonstrated, and the combinations of HC with traditional FLIM analysis were explained. We assessed HC methods with both simulated and experimental datasets. The results reveal that HC not only increases analysis speed (up to 106 times) but also enhances lifetime estimation accuracy. Fast lifetime analysis strategies were suggested with execution times around or below 30 *μ*s per histograms on MATLAB R2016a, 64-bit with the Intel Celeron CPU (2950M @ 2GHz).

## 1. Introduction

Fluorescence lifetime imaging (FLIM) [1] is a crucial technique for assessing microenvironments of fluorophores, such as pH, ${\mathrm{Ca}}^{2+}$, $O_{2}$, viscosity, or temperature [2–5]. Combining with Förster Resonance Energy Transfer (FRET) techniques [6], FLIM can be a powerful "quantum ruler" to measure protein conformations and interactions [7]. In contrast to fluorescence intensity imaging, FLIM is independent of fluorescence intensities and fluorophore concentrations, making FLIM a robust quantitative imaging technique for life sciences applications [8,9], medical diagnosis [10], drug developments [11,12], and flow diagnosis [13–15].

A fluorescence decay is usually modeled as a sum of exponential decay functions:

(1) $$f(t)=A\sum_{p=1}^{P}q_{p}\exp(-t/\tau_{p}),\sum_{p=1}^{P}q_{p}=1,$$

where A is the amplitude, $q_{p}$ and $\tau_{p}$ are the fraction and lifetime of the $p^{th}$ component, p=1,…,P. In vector forms, $q={\left[q_{1},\ldots ,q_{P}\right]}^{T}$ and $\tau ={\left[\tau_{1},\ldots ,\tau_{P}\right]}^{T}$. In reality, the measured signal is a convolution of f(t) and the instrument response function (IRF) irf(t),

(2) h(t)=irf(t)∗f(t)+ϵ(t),

where ϵ(t) is noise.

FLIM analysis is equivalent to solving the inverse problem from Eq. (2) with the measured h(t) to obtain q and τ. FLIM experiments can be conducted either in time- or frequency-domain manners [8]. In time-domain approaches, samples are illuminated with ultrashort laser pulses. h(t) is measured using a time-correlated single-photon counting (TCSPC) system [16,17] with photomultiplier tubes, delay line anode detectors [18] or single-photon avalanche diodes [19] in scanning or widefield modes. h(t) can also be measured with time-gated cameras [20,21] and streak cameras [22,23]. There are also frequency-domain approaches [24,25], but we will focus on time-domain approaches in this report.

A fluorescence decay histogram measured by a TCSPC system can be:

(3) $$h_{m}=\sum_{k=0}^{m}ir\;f_{k-m}\cdot f_{m}+\epsilon_{m},$$

where

(4) $$ir\;f_{m}=\int_{m\Delta t}^{(m+1)\Delta t}ir\;f(t)dt,f_{m}=\int_{m\Delta t}^{(m+1)\Delta t}f(t)dt,m=0,\ldots ,M-1.$$

M is the number of time-bins, and Δt is the bin width (the TCSPC’s temporal resolution). We can express Eq. (3) in a vector form with $h={\left[h_{0},\ldots ,h_{M-1}\right]}^{T}$, $ir\;f={\left[ir\;f_{0},\ldots ,ir\;f_{M-1}\right]}^{T}$, and $f={\left[f_{0},\ldots ,f_{M-1}\right]}^{T}$.

With h and irf already measured, A, q and τ can be extracted with a lifetime determination algorithm by solving a nonlinear minimization problem $\arg\min\|h-\hat{h}{\|}^{2}$, where $\hat{h}$ is the estimated histogram. The iterative convolution (IC) is commonly used with the least-squared method (LSM) [26,27] for solving the inverse problem, denoted as IC-LSM. Still, IC-LSM suffers from low photon efficiency and slow analysis. Several deconvolution approaches have been developed to enhance the analysis, such as the Laguerre expansion [28–30], the non-fitting and the global fitting [31,32] methods. The Laguerre expansion methods speed up deconvolution procedures by converting the nonlinear-fitting problem to a linear-fitting problem estimating a Laguerre basis set’s expansion coefficients. The non-fitting methods, including the centre-of-mass method (CMM) [33–35], the integral extraction method (IEM) [36,37], the phasor method [38,39], or the rapid lifetime determination method [40,41], can provide rapid average lifetime analysis [42]. The global fitting methods can accelerate analysis by changing the estimation mode from the pixel-wise mode to a global fitting mode and using spatial lifetime invariances of fluorescent species in an image to reduce the degree of freedom significantly. There are two strategies, IC [31] and the variable projection (VP) method [32], for implementing global fitting.

However, the Laguerre expansion, the non-fitting and the global fitting methods are not fast enough for growing demands for real-time FLIM. This work presents a histogram clustering (HC) method for improving FLIM analysis in analysis speed and accuracy. Section 2 (Methods) summarizes the workflows for decay parameter image reconstructions with and without HC. We will then introduce and demonstrate the HC method. Besides the algorithms used in this work, HC can also accelerate other algorithms, such as the maximum likelihood method [43], Bayesian methods [44], and deep-learning methods [45]. In Section 3 (Results and Analysis), synthetic and experimental TCSPC datasets will be used to evaluate the HC method’s performances. Suggestions of the fastest algorithms for different outcomes will be given.

## 2. Methods

### 2.1 Modes for decay parameter image reconstructions

**Fig. 1.**
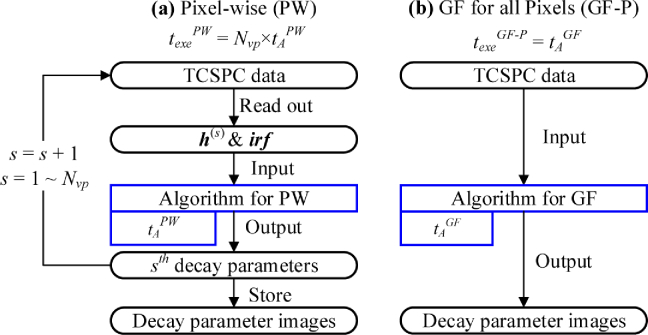
Flow diagrams of (a) the pixel-wise (PW) mode and (b) the global fitting mode for all pixels (GF-P).

Figure 1(a) shows the **P**ixel-**W**ise (PW) mode’s workflow. $N_{v\;p}$ is the number of valid pixels in a TCSPC dataset whose intensities are beyond a threshold. Histogram s, denoted as $h^{\left(s\right)}$, is sent into an algorithm for PW along with irf, $s=1,\ldots ,N_{v\;p}$. After $N_{v\;p}$ histograms are analyzed pixel-by-pixel, decay parameter images are produced. The total execution time $t_{exe}^{P\;W}=N_{v\;p}\times t_{A}^{P\;W}$, $t_{A}^{P\;W}$ is the adopted algorithm’s execution time for PW.

Figure 1(b) shows the workflow of the **G**lobal **F**itting mode for all **P**ixels, denoted as GF-P. Instead of estimating decay parameters individually for each pixel, GF-P assumes lifetimes τ are constants, and A and q vary across the image. $t_{exe}^{G\;F\;-P}=t_{A}^{G\;F}$. $t_{A}^{G\;F}$ is the adopted algorithm’s execution time for GF.

**Fig. 2.**
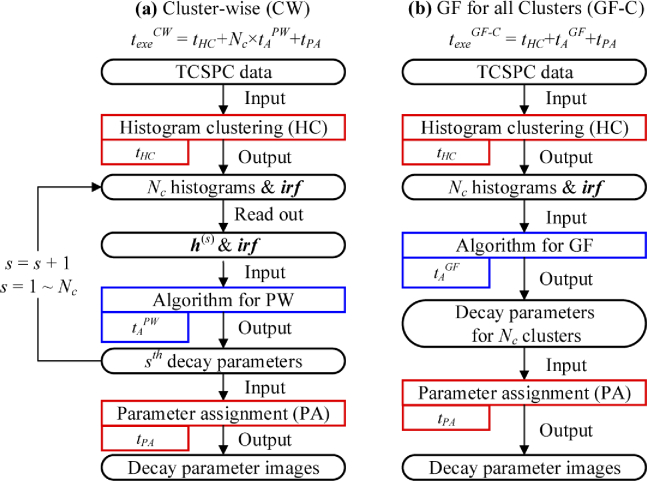
Flow diagrams of (a) the cluster-wise mode (CW) and (b) the global fitting mode for all clusters (GF-C).

Figure 2 shows the workflows where the HC method is embedded. Figure 2(a) shows the **C**luster-**W**ise (CW) mode, which combines PW and HC; likewise, Fig. 2(b) shows the **G**lobal **F**itting mode for all **C**lusters (GF-C), which combines GF-P and HC.

In CW, $N_{v\;p}$ histograms are first sorted by HC, whose execution time is $t_{HC}$, into $N_{c}$ classes with $N_{c}$ cluster-histograms ${\bar{h}}^{\left(s\right)}$, $s=1,\ldots ,N_{c}$. ${\bar{h}}^{\left(s\right)}$ is used to estimate decay parameters for Cluster s. Then, the decay parameters are assigned to the corresponding cluster’s pixels with a parameter assignment function, whose execution time is $t_{P\;A}$. Therefore, $t_{exe}^{C\;W}=t_{H\;C}+N_{c}\times t_{A}^{P\;W}+t_{P\;A}$.

In GF-C, $N_{v\;p}$ histograms are processed with HC first, and the output $N_{c}$ histograms are sent into an algorithm for GF. Decay parameters for all clusters are obtained and assigned to the pixels in corresponding clusters with the parameter assignment function. Therefore, $t_{exe}^{G\;F\;-C}=t_{H\;C}+t_{A}^{G\;F}+t_{P\;A}$.

The algorithms used in this work are reviewed in Supplement 1.

### 2.2 Histogram clustering

In reality, there are always many pixels within the field of view showing similar histogram profiles, and it’s unnecessary to analyze them individually because it would be time-consuming. The idea of HC is to sort histograms with similar profiles and to divide them into $N_{c}$ clusters. If histograms have similar decay profiles, they are supposed to show similar decay parameters. Therefore, we can average similar decay profiles in a cluster into one profile to estimate decay parameters and then assign them to all pixels. With this arrangement, we only need to process $N_{c}$ instead of $N_{v\;p}$ histograms. HC significantly speedups FLIM analysis.

**Fig. 3.**
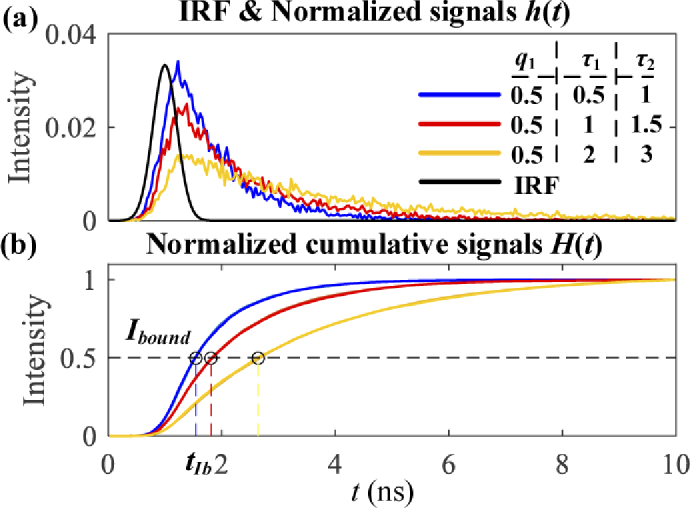
Illustrations of (a) an IRF and normalized signal profiles h(t) following a bi-exponential decay model and (b) cumulative signal profiles H(t).

For simplicity, we only discuss bi-exponential decays widely used in practice. Figure 3(a) shows an IRF and normalized signal profiles h(t) following a bi-exponential decay model, and Fig. 3(b) shows corresponding cumulative signals, $H(t)=\int_{0}^{t}h(t)dt$, which is not sensitive to Poisson noise due to the integration. Signal decay parameters are also labelled in Fig. 3(a). If we choose an intensity bound, $I_{bound}$, then each signal has a corresponding time delay, $t_{I\;b}$, to reach $I_{bound}$, as shown in Fig. 3(b).

**Fig. 4.**
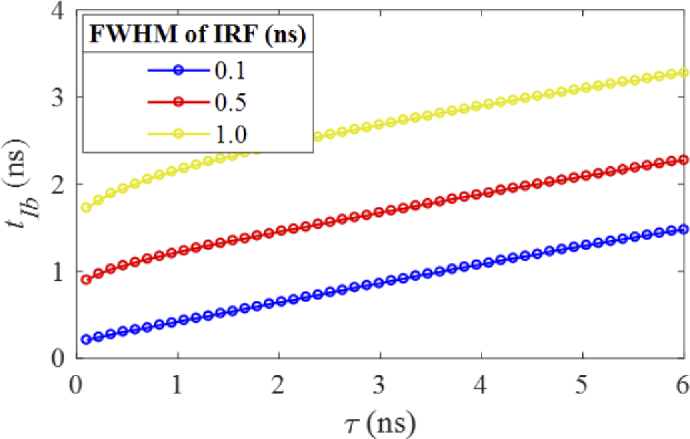
$t_{I\;b}$ of signals following mono-exponential models depending on τ under different irf(t).

It is straightforward for mono-exponential decays, f(t)=Aexp⁡(−t/τ), that $t_{I\;b}$ has an approximately linear relationship with τ. Figure 4 shows $t_{I\;b}$ curves with different IRFs which introduce time-shifts (assuming that IRFs for all histograms are the same in a scanning system). If a multichannel sensor is used, IRF alignments are required before using HC.

**Fig. 5.**
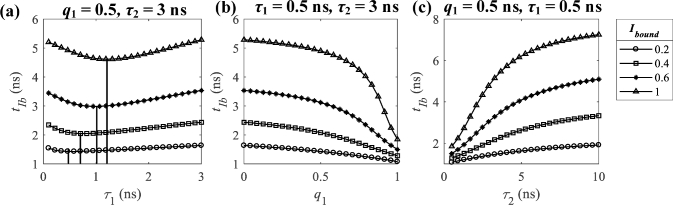
$t_{I\;b}$ for (a) Case A: $q_{1}$ = 0.5, $\tau_{1}$ = 0.1 ∼ 3 ns, and $\tau_{2}$ = 3 ns, (b) Case B: $q_{1}$ = 0 ∼ 1, $\tau_{1}$ = 0.5 ns, and $\tau_{2}$ = 3 ns, (c) Case C: $q_{1}$ = 0.5, $\tau_{1}$ = 0.5 ns, and $0.5\leq \tau_{2}\leq 10$ ns with different $I_{bound}$.

However, it is less straightforward for bi-exponential decays. Thus, we used numerical methods to conduct analysis, as shown in Fig. 5, in which three cases were simulated to explain how the proposed concept works. Case A has $q_{1}$ = 0.5, $0.1\leq \tau_{1}\leq 3$ ns, and $\tau_{2}$ = 3 ns as shown in Fig. 5(a); Case B has $0\leq q_{1}\leq 1$, $\tau_{1}$ = 0.5 ns, and $\tau_{2}$ = 3 ns as shown in Fig. 5(b); Case C has $q_{1}$ = 0.5, $\tau_{1}$ = 0.5 ns, and $0.5\leq \tau_{2}\leq 10$ ns as shown in Fig. 5(c). The IRF follows a Gaussian distribution with an FWHM of 0.5 ns.

For Case A, $t_{I\;b}$ is not monotonic with $\tau_{1}$, and the monotonic range and the slope are functions of $I_{bound}$. As $I_{bound}$ increases, the slope increases with a smaller monotonic range. The profiles with $\tau_{1}$ outside the range are wrongly sorted into a cluster with a larger $\tau_{1}$. The monotonic ranges for $I_{bound}$ = 0.2 and 0.6 are 0.4 ∼ 3 ns and 1 ∼ 3 ns, respectively. For Cases B and C, $t_{I\;b}$ is monotonic (decreasing and increasing) with $q_{1}$ and $\tau_{2}$ for all $I_{bound}$, respectively. For the signals like Cases A ∼ C (which only have one variable), we can cluster the signals by $t_{I\;b}$ with a proper $I_{bound}$ considering the monotonic range. For example, for Case A, if the shortest lifetime is around 0.5 ns, $I_{bound}$ = 0.2 is a proper choice; for Cases B and C, $I_{bound}$ can be set arbitrarily in 0.1 ∼ 1. We use $I_{bound}$ = 0.2 hereafter.

However, it is not realistic that the signals in a dataset have one variable and two constant decay parameters. For instance, in FRET-FLIM applications, donors without FRET have a constant lifetime and donors interacting with acceptors have shorter lifetimes due to FRET. Therefore, the short and long lifetimes, $\tau_{1}$ and $\tau_{2}$, are donors’ lifetimes with and without FRET, and $q_{1}$ is the portion of the donors undergoing FRET among all donors. $q_{1}$ and $\tau_{1}$ are variables depending on FRET efficiency.

**Fig. 6.**
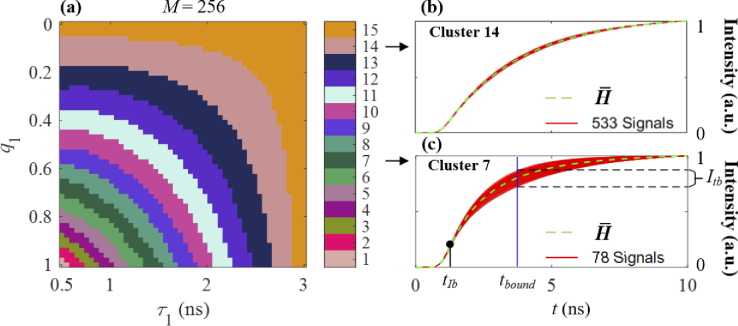
For Case D with M = 256, (a) clusters, cumulative signals (red solid lines) and averaged cumulative signals (green dash lines) for (b) Cluster 14 and (c) Cluster 7.

For FRET-FLIM datasets, such as Case D: $q_{1}$ = 0 ∼ 1, $\tau_{1}$ = 0.5 ∼ 3 ns, and $\tau_{2}$ = 3 ns, with two variables, it is not enough to divide the histograms only depending on $t_{I\;b}$, as histograms with different profiles would have the same $t_{I\;b}$ and be wrongly divided into one cluster. Figure 6(a) shows the resulting clusters ( $N_{1}$ = 15) in different colors for Case D with M = 256 depending on $t_{I\;b}$. Figures 6(b) and (c) show the cumulative signals in Clusters 14 and 7 in red, respectively, and the averaged cumulative histograms $\bar{H}$ for the clusters (green dash lines). When $q_{1}$ ∼ 0 (or $q_{1}$ ∼ 1) or $\tau_{1}$ ∼ $\tau_{2}$ (such as Clusters 14 and 15), the signals are nearly mono-exponential and have similar profiles, as shown in Fig. 6(b). However, the signals for other clusters (for example, Cluster 7) have the same $t_{I\;b}$, but the profiles after $t_{I\;b}$ diverge. At $t=t_{bound}$ in Fig. 6(c), the cumulative intensity $I_{t\;b}$ in Cluster 7 is within [0.7, 0.9]. Therefore, we can further divide each cluster into $N_{2}$ sub-clusters depending on $I_{t\;b}$.

Setting a larger $N_{2}$ can result in a higher clustering precision, which means histogram profiles in one cluster are more similar. Another way to increase clustering precision is interpolating h with M time-bins to $h^{inter\;p}$ with $M\cdot N_{inter\;p}$ time-bins. $N_{inter\;p}$ (≥ 1) is an interpolation factor, and $h^{inter\;p}$ can be expressed as

(5) $$h_{mN_{inter\;p}+n}^{inter\;p}=h_{m}/N_{inter\;p},n=0,\ldots ,N_{inter\;p}-1,m=0,\ldots ,M-1.$$

**Fig. 7.**
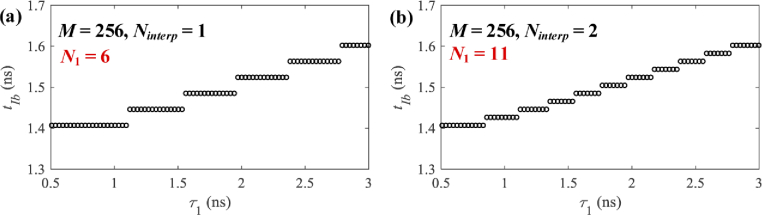
For Case A, clusters with (a) M = 256 and $N_{inter\;p}$ = 1 and (b) M = 256 and $N_{inter\;p}$ = 2.

Figure 7(a) shows the clusters for Case A with M = 256, $N_{inter\;p}$ = 1, and $N_{1}$ = 6. Figure 7(b) shows the clusters for M = 256, $N_{inter\;p}$ = 2, and $N_{1}$ = 11. The histograms in each cluster have a smaller range of $\tau_{1}$, leading to a higher clustering precision.

**Fig. 8.**
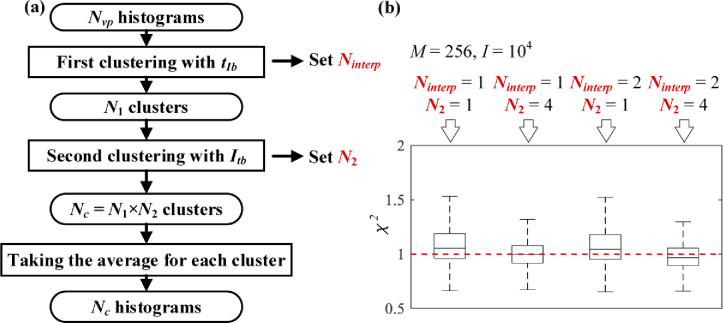
(a) Workflow of the HC method. (b) Boxplot of $\chi^{2}$ for Case D with different $N_{inter\;p}$ and $N_{2}$.

The HC workflow is summarized in Fig. 8(a). There are three steps: 1) depending on $t_{I\;b}$, $N_{v\;p}$ histograms are divided into $N_{1}$ clusters, which can be adjusted by setting $N_{inter\;p}$; 2) depending on $I_{tb}$, histograms in each of the $N_{1}$ clusters are further divided into $N_{2}$ sub-clusters, and $N_{c}=N_{1}\times N_{2}$ clusters are finally produced; 3) $N_{c}$ histograms are generated by obtaining the averaged histogram in each cluster.

To assess HC in terms of $N_{inter\;p}$ and $N_{2}$, we can define:

(6) $$\chi^{2(s)}=\frac{1}{M}\sum_{m=0}^{M-1}{\left|h_{m}^{\left(s\right)}-{\bar{h}}_{m}^{\left(s\right)}\right|}^{2}/h_{m}^{\left(s\right)},$$

where ${\bar{h}}_{m}^{\left(s\right)}$ is the cluster histogram produced with HC for the cluster including Pixel s. Figure 8(b) shows the boxplot of $\chi^{2(s)}$ for Case D for various combinations of $N_{inter\;p}$ and $N_{2}$. Poisson noise is included in each signal with a total intensity of $I=10^{4}$ photon counts. The higher $N_{inter\;p}$ and $N_{2}$ are, the lower $\chi^{2(s)}$ becomes, meaning higher accuracy. We set $N_{inter\;p}$ = 2 and $N_{2}$ = 4 for HC used in CW and GF-C modes in this work.

## 3. Results and analysis

Synthetic and experimental TCSPC datasets were used to assess the performances of HC. Table 1 summarizes different output types of algorithms: (1) the fitting method LE-LSM in PW and CW modes can produce q, τ, $\tau_{A}$, and $\tau_{I}$ images; (2) the non-fitting methods LE-IEM and CMM in PW and CW modes can produce $\tau_{A}$ and $\tau_{I}$ images; (3) the global fitting methods IC and VP in GF-P and GF-C modes can produce q, $\tau_{A}$, and $\tau_{I}$ images and constant τ. $\tau_{A}$ and $\tau_{I}$ are two types of average lifetimes, amplitude- and intensity-weighted average lifetimes,

(7) $$\tau_{A}=\sum_{p=1}^{P}q_{p}\tau_{p},\tau_{I}=\sum_{p=1}^{P}q_{p}\tau_{p}^{2}/\sum_{p=1}^{P}q_{p}\tau_{p}.$$

Using IEM to calculate $\tau_{A}$ requires conducting the deconvolution first. The Laguerre expansion method is employed for deconvolution when IEM is used; therefore, we denote the whole process as LE-IEM.

**Table 1.** Outputs of algorithms for different modes.

Mode	Algorithm		Output variables^*a*^			
			q	τ	$\tau_{I}$	$\tau_{A}$
Pixel-Wise (PW)/Cluster-Wise (CW)	Least-Squared Method with Laguerre Expansion	LE-LSM	I	I	I	I
	Integral Extraction Method with Laguerre Expansion	LE-IEM	X	X	X	I
	Centre-of-Mass Method	CMM	X	X	I	X
Global-Fitting for all Pixels (GF-P)/Global-Fitting for all Clusters (GF-C)	Iterative Convolution	IC	I	C	I	I
	Variable Projection	VP	I	C	I	I

^*a*^ Letters I and C represent that the outputs are images and constants, respectively.

Letter X stands for no output.

### 3.1 Simulated data

**Fig. 9.**
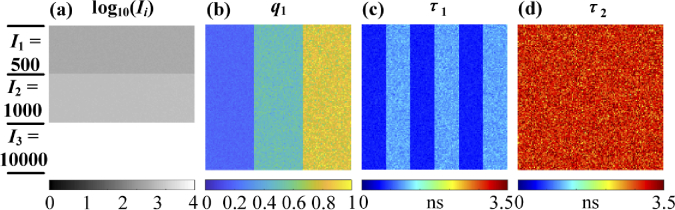
(a) $\log_{10}(I_{i})$, (b) $q_{1}$, (c) $\tau_{1}$, and (d) $\tau_{2}$ images of the synthetic TCSPC dataset.

The synthetic TCSPC dataset has an image size of 150 × 150 pixels and M = 256. The simulated signals are bi-exponential (P = 2). Figure 9(a) shows the $\log_{10}(I_{i})$ image consisting of three regions with integrated intensities of $I_{1}$ = 500, $I_{2}$ =1000, and $I_{3}$ = 10000, respectively. Possion noise is included in the dataset. Figures 9(b), 9(c), and 9(d) shows the $q_{1}$, $\tau_{1}$, and $\tau_{2}$ images, respectively. The $q_{1}$ image has three regions with mean values of [0.2, 0.5, 0.8] and relative standard deviations of 10%; the $\tau_{1}$ image has two regions with mean values of [0.5, 1] ns and relative standard deviations of 10%; the $\tau_{2}$ image has a mean value of 3 ns and a relative standard deviation of 10%.

The execution times ($t_{exe}$) and the mean squared errors (MSE) of the results evaluated by different algorithms without and with HC are summarized in Table 2. MSE is defined as:

(8) $$MSE=\sum_{s=1}^{N_{v\;p}}{\left|a^{\left(s\right)}-{\hat{a}}^{\left(s\right)}\right|}^{2}/N_{v\;p},$$

where $\hat{a}$ represents the estimated a ($a=q_{1},\tau_{1},\tau_{2},\tau_{A},\tau_{I}$). The results for the fitting and non-fitting methods in PW and CW modes and the global fitting methods in GF-P and GF-C modes are illustrated and analyzed in the following sections.

**Table 2.** $t_{exe}$ and MSE evaluated by algorithms without and with HC.

Mode	Algorithm	$t_{exe}$ (s)	MSE				
			$q_{1}$	$\tau_{1}$ (${\mathrm{ns}}^{2}$)	$\tau_{2}$ (${\mathrm{ns}}^{2}$)	$\tau_{A}$ (${\mathrm{ns}}^{2}$)	$\tau_{I}$ (${\mathrm{ns}}^{2}$)
Without HC							
PW	LE-LSM	389.45	0.019	0.173	0.198	0.110	0.027
	LE-IEM	62.30	X	X	X	0.102	X
	CMM	0.20	X	X	X	X	0.185
GF-P	IC	724.82	0.100	X	X	0.678	1.098
	VP	3.34	0.033	X	X	0.122	0.178
With HC							
CW	LE-LSM	3.36	0.011	0.102	0.104	0.037	0.025
	LE-IEM	0.63	X	X	X	0.038	X
	CMM	0.20	X	X	X	X	0.180
GF-C	IC	11.85	0.017	X	X	0.102	0.050
	VP	0.31	0.014	X	X	0.048	0.093

#### 3.1.1 Fitting method LE-LSM in PW and CW modes

**Fig. 10.**
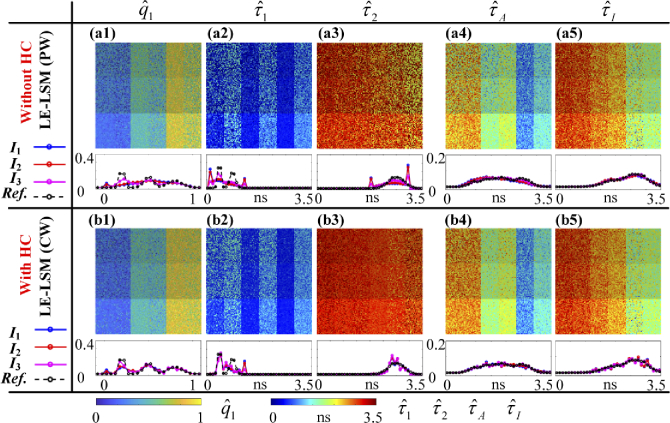
${\hat{q}}_{1}$, ${\hat{\tau }}_{1}$, ${\hat{\tau }}_{2}$, ${\hat{\tau }}_{A}$, and ${\hat{\tau }}_{I}$ images with LE-LSM in (a1) – (a5) PW (without HC) and (b1) – (b5) CW (with HC) modes. Histograms of different intensity regions (blue, red, and magenta for $I_{1}$, $I_{2}$, and $I_{3}$, respectively) and the true histogram (black dash line).

Figure 10 shows ${\hat{q}}_{1}$, ${\hat{\tau }}_{1}$, ${\hat{\tau }}_{2}$, ${\hat{\tau }}_{A}$, and ${\hat{\tau }}_{I}$ images estimated with LE-LSM in (a1) – (a5) PW (without HC) and (b1) – (b5) CW (with HC) modes, respectively. The pixel brightness of each image represents the intensity. The parameter histograms (${\hat{q}}_{1}$, ${\hat{\tau }}_{1}$, ${\hat{\tau }}_{2}$, ${\hat{\tau }}_{A}$, and ${\hat{\tau }}_{I}$) of different intensity regions (blue, red, and magenta lines for $I_{1}$, $I_{2}$, and $I_{3}$ respectively) and the true histogram (black dash line) are attached to each image. HC improves the estimated images, especially for Regions $I_{1}$ and $I_{2}$, as the histograms are closer to the truth than those estimated without HC. MSEs are reduced from 0.019 to 0.011 for $q_{1}$, from 0.173 ${\mathrm{ns}}^{2}$ to 0.102 ${\mathrm{ns}}^{2}$ for $\tau_{1}$, from 0.198 ${\mathrm{ns}}^{2}$ to 0.104 ${\mathrm{ns}}^{2}$ for $\tau_{2}$, from 0.110 ${\mathrm{ns}}^{2}$ to 0.037 ${\mathrm{ns}}^{2}$ for $\tau_{A}$, and from 0.027 ${\mathrm{ns}}^{2}$ to 0.025 ${\mathrm{ns}}^{2}$ for $\tau_{I}$. $t_{exe}$ is significantly reduced from 389.45 s to 3.36 s.

#### 3.1.2 Non-fitting methods LE-IEM and CMM in PW and CW modes

**Fig. 11.**
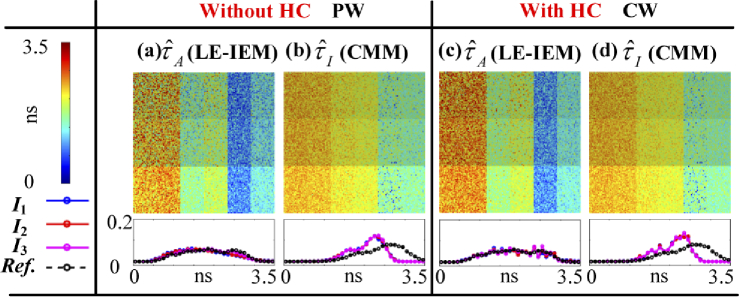
${\hat{\tau }}_{A}$ and ${\hat{\tau }}_{I}$ images and histograms produced by LE-IEM and CMM (a) – (b) without and (c) – (d) with HC.

Figure 11 shows ${\hat{\tau }}_{A}$ and ${\hat{\tau }}_{I}$ images and histograms produced by LE-IEM and CMM (a) – (b) without and (c) – (d) with HC. LE-IEM is for estimating ${\hat{\tau }}_{A}$. $MSE(\tau_{A})$ is improved from 0.102 ${\mathrm{ns}}^{2}$ to 0.038 ${\mathrm{ns}}^{2}$, and $t_{exe}$ is reduced from 62.30 s to 0.63 s with HC.

CMM is for estimating ${\hat{\tau }}_{I}$ with the shortest $t_{exe}$ either in PW or CW, around 0.20 s, but it has a bias, as shown in Figs. 11(b) and 11(d). $MSE(\tau_{I})$ is around 0.180 ${\mathrm{ns}}^{2}$. There is a way to correct the bias, as described in [46].

#### 3.1.3 Global fitting methods IC and VP in GF-P and GF-C modes

**Fig. 12.**
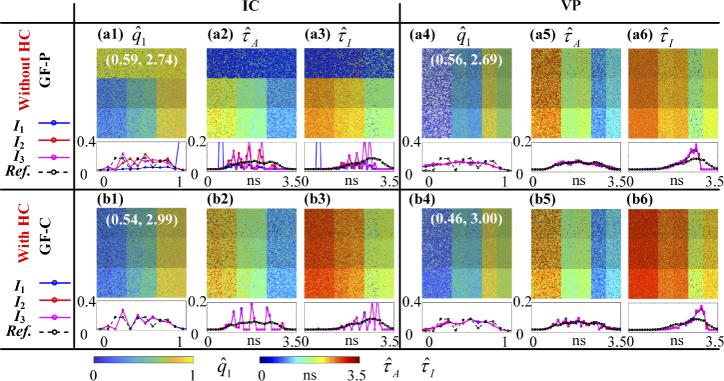
${\hat{q}}_{1}$, ${\hat{\tau }}_{A}$, and ${\hat{\tau }}_{I}$ images and histograms with (a1) – (a3) IC and (a4) – (a6) VP in GF-P mode and with (b1) – (b3) IC and (b4) – (b6) VP in GF-C mode. Constants (${\hat{\tau }}_{1}$, ${\hat{\tau }}_{2}$) are labelled in corresponding ${\hat{q}}_{1}$ images.

Figure 12 shows ${\hat{q}}_{1}$, ${\hat{\tau }}_{A}$, and ${\hat{\tau }}_{I}$ images estimated with (a1) – (a3) IC and (a4) – (a6) VP in GF-P (without HC) mode and with (b1) – (b3) IC and (b4) – (b6) VP in GF-C (with HC) mode. The estimated constants (${\hat{\tau }}_{1}$, ${\hat{\tau }}_{2}$) are labelled in corresponding ${\hat{q}}_{1}$ images. The estimations of $q_{1}$ in Region $I_{1}$ with IC in GF-P are mostly inaccurate, as shown in Fig. 12(a1) and (${\hat{\tau }}_{1}$, ${\hat{\tau }}_{2}$) = (0.59, 2.74) ns. As a result, ${\hat{\tau }}_{A}$ and ${\hat{\tau }}_{I}$ are also not correct in Region $I_{1}$, as shown in Figs. 12(a2) and (a3). In GF-C, IC performs better with a successfully estimated Region $I_{1}$, a significantly reduced $t_{exe}$ from 724.82 s to 11.85 s, and a reduced $MSE(q_{1})$ from 0.100 to 0.017, a reduced $MSE(\tau_{A})$ from 0.678 to 0.102, and a reduced $MSE(\tau_{I})$ from 1.098 to 0.050, as shown in Figs. 12(b1) - (b3).

HC also accelerates VP from 3.34 s to 0.31 s with a reduced $MSE(q_{1})$ from 0.033 to 0.014, a reduced $MSE(\tau_{A})$ from 0.122 to 0.048, and a reduced $MSE(\tau_{I})$ from 0.178 to 0.093, as shown in Figs. 12(b4) – (b6).

Although VP has some invalid estimations with ${\hat{q}}_{1}$ < 0 (pixels in white) when $q_{1}$ is small, as shown in Figs. 12(a4) and (b4), its ${\hat{\tau }}_{A}$ and ${\hat{\tau }}_{I}$ images are accurately evaluated without invalid pixels, as shown in Figs. 12(a5), (a6), (b5), and (b6). Thus, VP in GF-C is a promising choice for fast average lifetime estimations for its short execution time ($t_{exe}$ = 0.31 s).

In conclusion, HC not only accelerates analysis but also enhances accuracy (MSE). HC sorts histograms with similar profiles into a cluster and takes the average of histograms for lifetime determination, equivalent to increasing the number of photon counts and reducing noise. Therefore, the decay parameters estimated with the average cluster histogram by lifetime determination algorithms have higher accuracy than those of individual histograms. Although the decay parameters of the histograms in one cluster have a deviation from those estimated with the average cluster histogram, the results indicate that the error introduced by HC is smaller than that introduced by processing original histograms with a relatively lower photon count.

### 3.2 Experimental data

Mouse raw macrophage cells were routinely cultured in DMEM (Dulbecco’s Modified Eagle Medium) supplemented with 10% FCS (Fetal Calf Serum) under 5% ${\mathrm{CO}}_{2}$ at 37${\mathrm{37}}^{o}C$. Cells were seeded on glass cover slips in 24-well plates and cultured overnight for bacterial infection. Bacteria engineered to express GFP (Green Fluorescent Protein) were harvested from an early exponential phase and added to the cells with an MOI (Multiplicity of Infection) = 100. Cells were washed with PBS (Phosphate-Buffered Saline) and stained for actin with phalloidin Alexa Flour 546 (Thermo Fisher Scientific). The scanning FLIM used in this work is LSM510 (Carl Zeiss), equipped with a TCSPC module (SPC-830, Becker & Hickl GmbH). The sample was excited by a tunable femtosecond Ti: Sapphire laser (Chameleon, Coherent) at 850 nm as a two-photon excitation source. The repetition rate is 80 MHz, and the pulse width is less than 200 fs. The emitted photons were collected through a 63× water-immersion objective lens (N.A. = 1.0) and a 500 ∼ 550 nm bandpass filter and transferred into a photomultiplier tube.

**Fig. 13.**
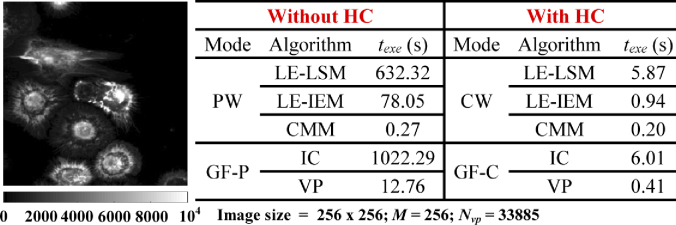
Intensity image and $t_{exe}$ for all algorithms without and with HC.

Figure 13 shows the intensity image and $t_{exe}$ for all algorithms without or with HC for a TCSPC dataset. The estimations of three output types are shown as follows.

#### 3.2.1 Type 1: ${\hat{q}}_{1}$, ${\hat{\tau }}_{1}$, and ${\hat{\tau }}_{2}$ images with LE-LSM in PW and CW modes

**Fig. 14.**
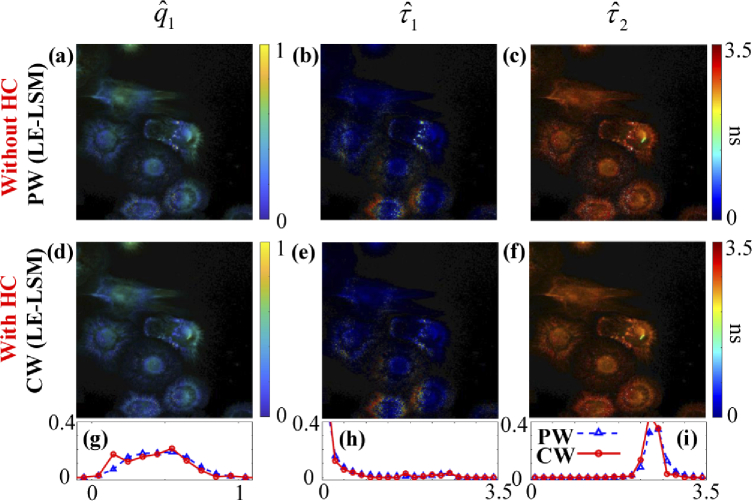
${\hat{q}}_{1}$, ${\hat{\tau }}_{1}$, and ${\hat{\tau }}_{2}$ images from LE-LSM (a) - (c) without and (d) – (f) with HC. Histograms of (g) ${\hat{q}}_{1}$, (h) ${\hat{\tau }}_{1}$, and (i) ${\hat{\tau }}_{2}$ in PW (blue) and CW (red) modes.

Figure 14 shows (a) ${\hat{q}}_{1}$, (b) ${\hat{\tau }}_{1}$, and (c) ${\hat{\tau }}_{2}$ images with LE-LSM in PW, (d) - (f) the results with LE-LSM in CW, and the histograms of (g) ${\hat{q}}_{1}$, (h) ${\hat{\tau }}_{1}$, and (i) ${\hat{\tau }}_{2}$ in PW (blue) and CW (red) modes. LE-LSM shows similar lifetime estimation performances in PW and CW, whereas LE-LSM in CW ($t_{exe}$ = 5.87 s) is faster than LE-LSM in PW ($t_{exe}$ = 632.32 s). Therefore, LE-LSM used in CW is a better choice for Type 1.

#### 3.2.2 Type 2: ${\hat{q}}_{1}$ image and constants (${\hat{\tau }}_{1}$, ${\hat{\tau }}_{2}$) with IC and VP in GF-P and GF-C modes

**Fig. 15.**
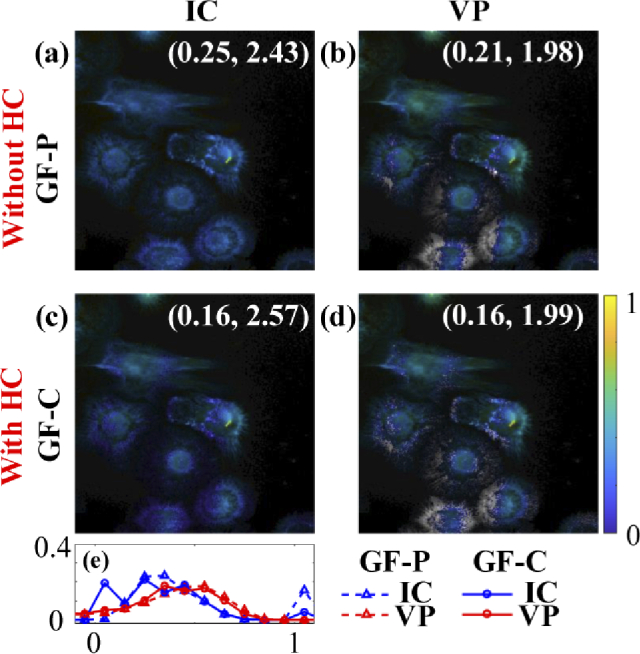
${\hat{q}}_{1}$ images from IC and VP (a) – (b) without and (c) – (d) with HC. (e) Histograms of ${\hat{q}}_{1}$ with IC (dash blue) and VP (dash red) in GF-P and IC (solid blue) and VP (solid red) in GF-C.

Figure 15 shows ${\hat{q}}_{1}$ images with (a) IC and (b) VP in GF-P, (c) IC and (d) VP in GF-C. Figure 15(e) shows histograms of ${\hat{q}}_{1}$ with IC (dash blue) and VP (dash red) in GF-P and IC (solid blue) and VP (solid red) in GF-C. The constants (${\hat{\tau }}_{1}$, ${\hat{\tau }}_{2}$) of each approach are attached in ${\hat{q}}_{1}$ images.

#### 3.2.3 Type 3: ${\hat{\tau }}_{A}$ and ${\hat{\tau }}_{I}$ images

**Fig. 16.**
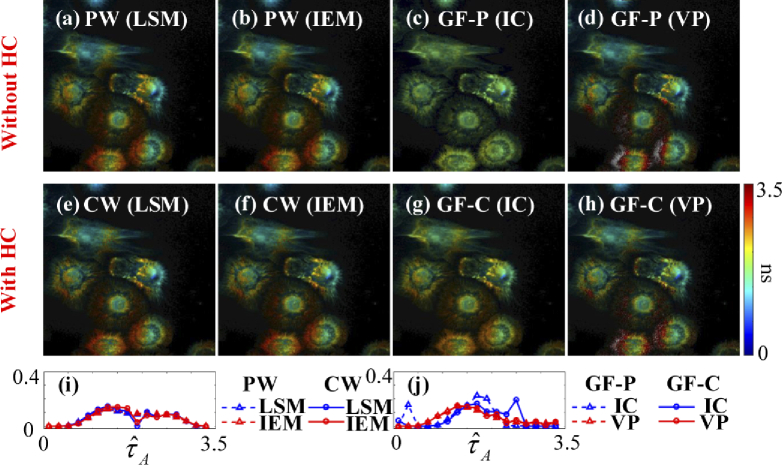
${\hat{\tau }}_{A}$ images from the algorithms (a) – (d) without and (e) – (h) with HC. (i) Histograms of ${\hat{\tau }}_{A}$ with LE-LSM and LE-IEM in PW and CW. (j) histograms of ${\hat{\tau }}_{A}$ with IC and VP in GF-P and GF-C.

**Fig. 17.**
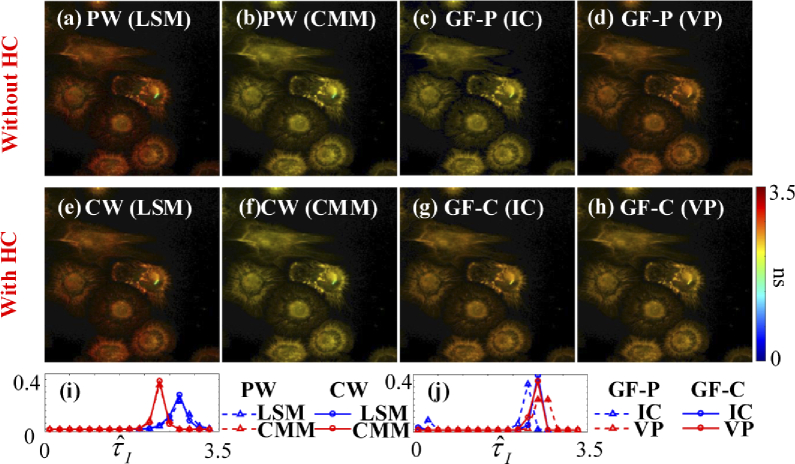
${\hat{\tau }}_{I}$ images from the algorithms (a) – (d) without and (e) – (h) with HC. (i) Histograms of ${\hat{\tau }}_{I}$ with LE-LSM and CMM in PW and CW. (j) histograms of ${\hat{\tau }}_{I}$ with IC and VP in GF-P and GF-C.

Figure 16 shows ${\hat{\tau }}_{A}$ images (a) – (d) without and (e) – (h) with HC. Figure 16(i) shows the histograms of ${\hat{\tau }}_{A}$ with LE-LSM and LE-IEM in PW and CW. Figure 16(j) shows the histograms of ${\hat{\tau }}_{A}$ with IC and VP in GF-P and GF-C. Figure 17 shows ${\hat{\tau }}_{I}$ images (a) – (d) without and (e) – (h) with HC. Figure 17(i) shows the histograms of ${\hat{\tau }}_{I}$ with LE-LSM and CMM in PW and CW. Figure 17(j) shows the histograms of ${\hat{\tau }}_{I}$ with IC and VP in GF-P and GF-C.

Like the conclusions drawn from simulations, LE-LSM in CW is the fastest for Type 1 with $t_{exe}$ = 5.87 s; VP in GF-C is the fastest for Type 2 with $t_{exe}$ = 0.41 s. For average lifetime images, VP in GF-C is the fastest for both $\tau_{A}$ and $\tau_{I}$ with $t_{exe}$ = 0.41 s, LE-IEM in CW is the second one for $\tau_{A}$ with $t_{exe}$ = 0.94 s; meanwhile, CMM in CW is the fastest for $\tau_{I}$ with $t_{exe}$ = 0.20 s.

## 4. Conclusion

We developed a histogram clustering (HC) method to accelerate FLIM analysis. HC can improve both the speed and the accuracy for FLIM analysis by sorting histograms with similar profiles in a dataset into several clusters and significantly reducing the number of histograms to be analyzed. The HC method implements clustering with two features of a histogram. Several commonly used lifetime determination algorithms’ performances for producing decay parameter images without and with HC were compared using synthetic and experimental datasets. For different output types, the fastest FLIM analysis methods are suggested: 1) LE-LSM with HC for all lifetime component images with an execution time ($t_{exe}$) of 5.87 s, 106-fold shorter than $t_{exe}$ without HC; 2) VP with HC for constant lifetimes, $q_{1}$, $\tau_{A}$, and $\tau_{I}$ images with $t_{exe}$ = 0.41 s, 32-fold shorter than $t_{exe}$ without HC; 3) LE-IEM with HC as the second choice for $\tau_{A}$ with $t_{exe}$ = 0.94 s, 78-fold shorter than $t_{exe}$ without HC, and CMM as the second choice for $\tau_{I}$ with $t_{exe}$ = 0.2 s without or with HC (biased if the largest lifetime > T/4). The analysis was conducted in Matlab, and it can be translated to C or other environments to speed up the analysis. We believe the proposed HC method can benefit applications demanding real-time FLIM such as clinical diagnosis and fast screening.

## Data Availability

Data underlying the results presented in this paper are not publicly available and can be made available by the authors without undue reservation.

## Supplemental document

See Supplement 1 for supporting content.
