# Supplementary material for: Histogram clustering for rapid time-domain fluorescence lifetime image analysis
Source: Biomed Opt Express. 2021 Jun 21;12(7):4293–307. doi: 10.1364/BOE.427532 (PMC8367240; doi:10.1364/BOE.427532)
Supplement: Supplementary file 1 [file boe-12-7-4293-s001.pdf]

Supplemental Document

Biomedical Optics EXPRESS

Histogram clustering for rapid time-domain fluorescence lifetime image analysis: supplement

YAHUI LI,[1,2,6] NATAKORN SAPERMSAP,[3] JUN YU,[4] JINSHOU TIAN,[1,2] YU CHEN,[3] AND DAVID DAY-UEI LI[5,7]

[1] *Key Laboratory of Ultra-fast Photoelectric Diagnostics Technology, Xi'an Institute of Optics and Precision Mechanics, Xi'an Shaanxi 710049, China*
[2] *Collaborative Innovation Center of Extreme Optics, Shanxi University, Taiyuan Shanxi 030006, China*
[3] *Department of Physics, Scottish Universities Physics Alliance, University of Strathclyde, Glasgow, G4 0NG, United Kingdom*
[4] *Strathclyde Institute of Pharmacy and Biomedical Sciences, University of Strathclyde, Glasgow G4 0RE, United Kingdom*
[5] *Department of Biomedical Engineering, University of Strathclyde, Glasgow G1 0NW, United Kingdom*
[6] *yahuili777@hotmail.com*
[7] *David.Li@strath.ac.uk*

This supplement published with The Optical Society on 21 June 2021 by The Authors under the terms of the Creative Commons Attribution 4.0 License in the format provided by the authors and unedited. Further distribution of this work must maintain attribution to the author(s) and the published article's title, journal citation, and DOI.

Supplement DOI: https://doi.org/10.6084/m9.figshare.14748114

Parent Article DOI: https://doi.org/10.1364/BOE.427532

Histogram clustering for rapid time-domain fluorescence lifetime imaging analysis: supplemental document

1. PIXEL-WISE LIFETIME DETERMINATION ALGORITHMS

A. Fitting methods

Fitting lifetime determination algorithms for the pixel-wise (PW) mode solve a minimization problem for *Histogram s*,

$$[\hat{A}^{(s)}, \hat{\boldsymbol{q}}^{(s)}, \hat{\boldsymbol{\tau}}^{(s)}] = \arg\min \|\boldsymbol{h}^{(s)} - \hat{\boldsymbol{h}}^{(s)}\|^2, \tag{S1}$$

where $\hat{\boldsymbol{h}}^{(s)}$ denotes the estimated histogram for $\boldsymbol{h}^{(s)}$, $\|.\|$ stands for the l_2 vector norm. $\hat{A}^{(s)}$, $\hat{\boldsymbol{q}}^{(s)}$, and $\hat{\boldsymbol{\tau}}^{(s)}$ can be obtained by solving Problem (S1).

A.1. Laguerre expansion

The Laguerre expansion (LE) method estimates the underlying fluorescence decay $f(s)$ with an ordered set of discrete-time Laguerre basis functions (LBFs) [1],

$$\hat{f}_m^{(s)} = \sum_{l=0}^{L-1} \hat{c}_l^{(s)} b_l(m;\alpha), \tag{S2}$$

where L and α are the basis parameters and $\hat{c}_l^{(s)}$ is the estimated l^{th} expansion coefficient of Histogram s. The lth discrete-time LBF is defined as

$$b_l(m;\alpha) = \alpha^{(m-l)/2}(1-\alpha)^{1/2}\sum_{i=0}^{l}(-1)^i \begin{pmatrix} m \\ i \end{pmatrix}\begin{pmatrix} l \\ i \end{pmatrix} \alpha^{l-i}(1-\alpha)^i, \tag{S3}$$

where $l = 0, \ldots, L-1$, and $0 < \alpha < 1$. With Eq. (S2), the estimated signal becomes

$$\hat{h}_m^{(s)} = \sum_{i=0}^{m}\sum_{l=0}^{L-1} \hat{c}_l^{(s)} \cdot irf_{m-i} \cdot b_l(i;\alpha) + \epsilon_m = \sum_{l=0}^{L-1} \hat{c}_l^{(s)} \cdot v_l(m;\alpha) + \epsilon_m, \tag{S4}$$

where $v_l(m;\alpha) = \sum_{l=0}^{m} irf_{m-i} \cdot b_l(i;\alpha)$.

Then, Problem (S1) becomes,

$$\hat{\boldsymbol{c}}^{(s)} = \arg\min \|\boldsymbol{h}^{(s)} - \boldsymbol{V}\hat{\boldsymbol{c}}^{(s)}\|^2, \tag{S5}$$

where $V = [\boldsymbol{v}_0, \ldots, \boldsymbol{v}_{L-1}]$, $\boldsymbol{v}_l = [v_l(0;\alpha), \cdots, v_k(M-1;\alpha)]^T$, and $\hat{\boldsymbol{c}}^{(s)} = \left[\hat{c}_0^{(s)}, \ldots, \hat{c}_{L-1}^{(s)}\right]^T$. Eq. (S5) can be addressed with the ordinary and constrained least-squared methods (LSM), as demonstrated in [2]. Setting proper L and α depends on the lifetime dynamic range and the measurement window $T = M\Delta t$. To guarantee that the estimated decay physically agrees with the real decay, we adopted the constrained LSM with $L = 16$ and $\alpha = 0.912$ in this work as suggested in [1] to ensure robust analysis for 0.5 ns $< \tau_p <$ 3 ns, $\Delta t = 0.039$ ns and $T = 10$ ns.

Once $\hat{\boldsymbol{c}}^{(s)}$ is determined, $\hat{\boldsymbol{f}}^{(s)}$ can be recovered with Eq. (S2). Then decay parameters of Histogram s can be extracted from $\hat{\boldsymbol{f}}^{(s)}$ using fitting methods, such as LSM and the maximum likelihood method or non-fitting methods, to be discussed in the next section.

B. Non-fitting methods

Non-fitting methods, including the centre-of-mass method (CMM), the integral extraction method (IEM), the phasor method (Phasor) and the rapid lifetime determination method (RLD), determine average lifetimes for $\boldsymbol{h}^{(s)}$. Two types of average lifetimes are generally useful for Förster resonance energy transfer and dynamic quenching applications [3]. They are the intensity-weighted lifetime τ_I and the amplitude-weighted lifetime τ_A [4, 5],

$$\tau_I = \sum_{p=1}^{P} q_p \tau_p^2 / \sum_{p=1}^{P} q_p \tau_p, \tau_A = \sum_{p=1}^{P} q_p \tau_p. \tag{S6}$$

The average lifetimes determined by CMM and IEM turn out to be τ_I and τ_A, respectively. The outcomes are close to τ_I from Phasor and are neither τ_I nor τ_A from RLD [3]. Therefore, we only focus on CMM and IEM in this work.

B.1. CMM

The average lifetime evaluated with CMM can be expressed as

$$\begin{aligned} \tau_{CMM}^{(s)} &= \frac{\int_0^\infty t \cdot h^{(s)}(t)dt}{\int_0^\infty h^{(s)}(t)dt} - \frac{\int_0^\infty t \cdot irf(t)dt}{\int_0^\infty irf(t)dt} = \frac{\sum_{p=1}^{P} q_p^{(s)} \tau_p^{(s)2}}{\sum_{p=1}^{P} q_p^{(s)} \tau_p^{(s)}} \\ &\approx \frac{\sum_{m=0}^{M-1} t_m \cdot h_m^{(s)}}{\sum_{m=0}^{M-1} h_m^{(s)}} - \frac{\sum_{m=0}^{M-1} t_m \cdot irf_m}{\sum_{m=0}^{M-1} irf_m} \end{aligned}, \tag{S7}$$

which is equal to $\tau_I^{(s)}$.

B.2. IEM

For IEM, deconvolution is required to obtain $\hat{\boldsymbol{f}}^{(s)}$ with which the average lifetime can be determined as

$$\begin{aligned} \tau_{IEM}^{(s)} &= -\frac{\int_0^\infty g^{(s)}(t)dt}{\int_0^\infty g^{(s)\prime}(t)dt} \approx \sum_{P=1}^{P} q_p^{(s)} \tau_p^{(s)} \\ &\approx -\frac{\sum_{m=0}^{M-1} S_m \cdot \hat{f}_m^{(s)}}{\sum_{m=0}^{M-1} \frac{\hat{f}_m^{(s)} - \hat{f}_{m-1}^{(s)}}{\Delta t}} = -\frac{\Delta t \sum_{m=0}^{M-1} S_m \cdot \hat{f}_m^{(s)}}{\hat{f}_{M-1}^{(s)} - \hat{f}_0^{(s)}}, \end{aligned} \tag{S8}$$

where $S_m = [1/3, 4/3, 2/3, \ldots, 4/3, 1/3]$ are the coefficients for numerical integration based on Simpson's rule,

$$g^{(s)}(t) = A^{(s)} \sum_{p=1}^{P} q_p^{(s)} \tau_p^{(s)} e^{-\frac{t}{\tau_p}} \left[1 - e^{-\frac{\Delta t}{\tau_p}}\right]. \tag{S9}$$

$\tau_{IEM}^{(s)}$ is an estimator for $\tau_A^{(s)}$. In this work, $\hat{\boldsymbol{f}}^{(s)}$ is extracted with LE; therefore, we denote the whole process as LE-IEM.

2. GLOBAL-FITTING LIFETIME DETERMINATION ALGORITHMS

Algorithms for global-fitting (GF) construct a minimization problem for all histograms,

$$[\hat{A}^{(s)}, \hat{\boldsymbol{q}}^{(s)}, \hat{\boldsymbol{\tau}}] = \arg\min \sum_s \|\boldsymbol{h}^{(s)} - \hat{\boldsymbol{h}}^{(s)}\|^2, \tag{S10}$$

Solving Problem (S10), $\hat{A}^{(s)}$ and $\hat{\boldsymbol{q}}^{(s)}$ for Histogram s and constant lifetimes $\hat{\boldsymbol{\tau}}$ for all histograms can be estimated. There are two strategies, the iterative convolution (IC) method and the variable projection (VP) method, for implementing GF, i.e. addressing Eq. (S10). VP appears to be faster than IC, as investigated in [6].

A. Iterative convolution

The decay is estimated with

$$\hat{f}_m^{(s)} = \hat{A}^{(s)} \sum_{p=1}^{P} \hat{q}_p^{(s)} e^{-t_m/\hat{\tau}_p}, \tag{S11}$$

where $\hat{\tau}_p$ are estimated constant lifetimes for all histograms with $\hat{A}^{(s)}$ and $\hat{q}_p^{(s)}$ being the parameters for Histogram s.

Then the estimated signal can be expressed as

$$\hat{h}_m^{(s)} = \sum_{k=0}^{m} irf_{k-m} \cdot \hat{f}_m^{(s)}, m = 0, 1, 2, \ldots, M-1. \tag{S12}$$

With Eq. (S12), we can solve Eq. (S10) with constrained LSM. The analysis speed of IC is significantly affected by the chosen initial conditions. S. Pelet *et al*. introduced different approaches for initial conditions [7]. We adopted the lifetime segmentation approach as they suggested. The implementation was performed with the MATLAB code developed in [7].

B. Variable projection

The idea of the global fitting with VP is to minimize a projection function that depends only on nonlinear parameters $\boldsymbol{\tau}$, and obtain linear parameters $A^{(s)}$ and $\boldsymbol{q}^{(s)}$. A matrix whose columns only depend on $\boldsymbol{\tau}$ is constructed,

$$\boldsymbol{\Phi}(\hat{\boldsymbol{\tau}}) = [\boldsymbol{\varphi}_1(\hat{\tau}_1), \ldots, \boldsymbol{\varphi}_P(\hat{\tau}_P)], \tag{S13}$$

where $\hat{\boldsymbol{\tau}} = [\hat{\tau}_1, \ldots, \hat{\tau}_P]^T, \boldsymbol{\varphi}_p(\hat{\tau}_p) = [\varphi_p(\hat{\tau}_p; t_0), \ldots, \varphi_p(\hat{\tau}_p; t_{M-1})]^T$, and $\varphi_p(\hat{\tau}_p; t_m) = \sum_{k=0}^{m} irf_{k-m} \cdot \exp(-t_m/\hat{\tau}_p), p = 1, \ldots, P$.

Then the estimated signal can be written as

$$\hat{\boldsymbol{h}}^{(s)} = \boldsymbol{\Phi}\hat{\boldsymbol{a}}^{(s)}, \tag{S14}$$

where $\hat{\boldsymbol{a}}^{(s)} = \left[\hat{a}_1^{(s)}, \ldots, \hat{a}_P^{(s)}\right]^T, \hat{a}_p^{(s)} = \hat{A}^{(s)}\hat{q}_p^{(s)}$, and $\hat{h}_m^{(s)} = \sum_{p=1}^{P} \hat{a}_p^{(s)} \varphi_p(\hat{\tau}_p; t_m)$.

With the given notation, we can rewrite Problem (S10) as

$$[\hat{\boldsymbol{a}}, \hat{\boldsymbol{\tau}}] = \arg\min \sum_{s=1}^{N_{vp}} \left\| \boldsymbol{h}^{(s)} - \boldsymbol{\Phi}(\hat{\boldsymbol{\tau}})\hat{\boldsymbol{a}}^{(s)} \right\|^2. \tag{S15}$$

For a given set of $\hat{\boldsymbol{\tau}}$, Eq. (S14)'s solution is $\hat{\boldsymbol{a}}^{(s)} = \boldsymbol{\Phi}^{-}(\hat{\boldsymbol{\tau}})\boldsymbol{h}^{(s)}$, where $\boldsymbol{\Phi}^{-}(\hat{\boldsymbol{\tau}})$ is the symmetric generalized inverse of $\boldsymbol{\Phi}(\hat{\boldsymbol{\tau}})$. Then Problem (S15) can be expressed as

$$\hat{\boldsymbol{\tau}} = \arg\min \sum_{s=1}^{N_{\text{vp}}} \left\| \boldsymbol{P}^{\perp}_{\boldsymbol{\Phi}(\hat{\boldsymbol{\tau}})} \boldsymbol{h}^{(s)} \right\|^2, \tag{S16}$$

where $\boldsymbol{P}^{\perp}_{\boldsymbol{\Phi}(\hat{\boldsymbol{\tau}})} = \boldsymbol{I} - \boldsymbol{\Phi}(\hat{\boldsymbol{\tau}})\boldsymbol{\Phi}^{-}(\hat{\boldsymbol{\tau}})$ [6]. Once $\hat{\boldsymbol{\tau}}$ is obtained from Eq. (S16), the linear parameter $\hat{\boldsymbol{a}}$ can be obtained as a solution of $\boldsymbol{h}^{(s)} = \boldsymbol{\Phi}\hat{\boldsymbol{a}}^{(s)}$. The implementation of VP is based on a modified version of the VARP2 code [8].

REFERENCES

1. Y. Zhang, Y. Chen, and D. D.-U. Li, "Optimizing Laguerre expansion based deconvolution methods for analyzing bi-exponential fluorescence lifetime images," Opt. Express **24**, 13894 (2016).
2. J. Liu, Y. Sun, J. Qi, and L. Marcu, "A novel method for fast and robust estimation of fluorescence decay dynamics using constrained least-squares deconvolution with Laguerre expansion," Phys. Medicine Biol. **57**, 843–865 (2012).
3. Y. Li, S. Natakorn, Y. Chen, M. Safar, M. Cunningham, J. Tian, and D. D.-U. Li, "Investigations on Average Fluorescence Lifetimes for Visualizing Multi-Exponential Decays," Front. Phys. **8**, 576862 (2020).
4. E. Fišerová and M. Kubala, "Mean fluorescence lifetime and its error," J. Lumin. **132**, 2059–2064 (2012).
5. A. Sillen and Y. Engelborghs, "The Correct Use of "Average" Fluorescence Parameters," Photochem. Photobiol. **67**, 475–486 (1998).
6. S. C. Warren, A. Margineanu, D. Alibhai, D. J. Kelly, C. Talbot, Y. Alexandrov, I. Munro, M. Katan, C. Dunsby, and P. M. W. French, "Rapid Global Fitting of Large Fluorescence Lifetime Imaging Microscopy Datasets," PLoS ONE **8**, e70687 (2013).

7. S. Pelet, M. Previte, L. Laiho, and P. C. So, "A Fast Global Fitting Algorithm for Fluorescence Lifetime Imaging Microscopy Based on Image Segmentation," Biophys. J. **87**, 2807–2817 (2004).
8. G. H. Golub and R. J. LeVeque, "Extensions and Uses of the Variable Projection Algorithm for Solving Nonlinear Least Squares Problems," in *Proceedings of the 1979 Army Numerical Analysis and Computers Conference.*, (Washington, DC., 1979), pp. 1–12.
